# Association between eosinophil count and prognosis in chronic obstructive pulmonary disease patients

**DOI:** 10.3389/fmed.2025.1525709

**Published:** 2025-09-03

**Authors:** Wan-Hsuan Hsu, Bo-Wen Shiau, Chih-Cheng Lai, Kuang-Ming Liao

**Affiliations:** ^1^Department of Internal Medicine, Chi Mei Medical Center, Tainan, Taiwan; ^2^Division of Hospital Medicine, Department of Internal Medicine, Chi Mei Medical Center, Tainan, Taiwan; ^3^School of Medicine, College of Medicine, National Sun Yat-sen University, Kaohsiung, Taiwan; ^4^Department of Internal Medicine, Chi Mei Medical Center, Chiali, Taiwan

**Keywords:** eosinophils, COPD, acute exacerbation, acute respiratory failure, mortality

## Abstract

**Background:**

The association between blood eosinophil levels and the risk of increased exacerbations, acute respiratory failure (ARF) and mortality in chronic obstructive pulmonary diseases (COPD) remains controversial. This association may predict future exacerbations and clinical outcomes among patients with COPD. This study aimed to clarify the relationship between eosinophil count and risk of acute exacerbation, ARF and mortality s in patients with COPD.

**Methods:**

The data for this retrospective cohort study were collected from TriNetX, a global multicentre research database. Using the Global Collaborative Research Networks, patients aged > 40 years with a diagnosis of COPD were examined for the association between blood eosinophil counts and the risk of acute exacerbation during a 3-year follow-up period. Cox proportional hazard models and Kaplan–Meier analysis were used to assess the risk of acute exacerbation between the non-eosinophilic (blood eosinophil counts <300 cells/μL) and eosinophilic groups (≥300 cells/μL) after propensity score matching. Other outcomes including survival and ARF, were also examined.

**Results:**

The non-eosinophilic group had a 1.27-fold lower risk of acute exacerbations of COPD (hazard ratio [HR], 0.790; 95% confidence interval [95% CI], 0.74–0.85; *p* < 0.001), a 1.22-fold lower risk of all-cause mortality (HR, 0.818; 95% CI, 0.76–0.88; *p* < 0.001) and a 1.39-fold lower risk of ARF (HR, 0.721; 95% CI, 0.66–0.78; *p* < 0.001) compared to the eosinophilic group during the 3-year follow-up period.

**Conclusion:**

Increased eosinophil count may be linked to an elevated risk of exacerbation, respiratory failure, and mortality in COPD.

## Introduction

Chronic obstructive pulmonary disease (COPD) is a chronic systemic inflammatory disease. The primary treatment goal in the management of COPD is to alleviate symptoms and minimize the frequency of acute exacerbations. According to the 2023 Global Initiative for Chronic Obstructive Lung Disease guidelines (1), previous exacerbations and blood eosinophils are key considerations in determining the appropriateness of initiating inhaled corticosteroid (ICS) therapy (2, 3).

The effectiveness of additional ICS therapy in patients experiencing acute exacerbation of COPD depends on the blood eosinophil counts. For patients with eosinophil counts below 100 cells/μL, introducing ICS generally exhibits limited impact. In contrast, patients with eosinophil counts of 300 cells/μL or higher are likely to benefit from the ICS use. For patients with eosinophil counts between 100 and 300 cells/μL, a gradual spectrum of treatment effectiveness is observed (4, 5). Within this range, certain individuals may experience a reduction in the frequency of acute exacerbations with the incorporation of ICS. Therefore, decisions regarding the prescription of ICS in patients with COPD should be guided by the individual’s historical trend of eosinophil count.

In a prior prospective observational study, 99 patients experiencing exacerbation of COPD were enrolled and grouped according to blood eosinophil count as low (<50 cells/μL), normal (50–150 cells/μL), or high (>150 cells/μL) in their study. The study revealed that the infection rates declined with increasing blood eosinophil counts. However, respiratory failure need for non-invasive or mechanical ventilation (MV) was similar across all eosinophil groups, and no statistically significant difference was observed in terms of in-hospital mortality. Notably, the study identified a significant reduction in the one-year survival rate following hospital discharge in the low eosinophil group compared to the high eosinophil group (6).

In a study conducted by Csomas et al., the risk of recurrence of moderate or severe exacerbation between eosinophilic and non-eosinophilic groups in COPD was examined, and no significant difference was identified (7). A separate investigation involving 493 patients hospitalized for acute COPD exacerbation revealed that patients with lower eosinophil counts had a higher incidence of respiratory failure and worse clinical outcomes compared to those with higher eosinophil counts (8).

Therefore, the association between eosinophil count and the risk of acute exacerbation of COPD remains controversial. Comprehensive nationwide studies elucidating the relationship between baseline eosinophil levels, the risk of acute exacerbation, and subsequent clinical outcomes in patients with COPD are lacking. The potential utility of employing clearly defined categories for eosinophil counts, specifically, low (<100 cells/μL), middle (100–299 cells/μL), and high (≥300 cells/μL), as per current guidelines to predict the risk of acute exacerbations in COPD has not been investigated. Distinct blood eosinophil levels in patients with COPD have been hypothesized to serve as predictors of future exacerbation risks and clinical outcomes.

## Materials and methods

### Data source

This retrospective cohort study used data from TriNetX, a global clinical research platform. This platform aggregates real-time de-identified electronic medical records from over 250 million patients across 19 countries, sourced from more than 120 healthcare organizations (HCOs) (9). This database encompasses a wide array of patient information, including diagnoses, procedures, medications, laboratory results, and genomic data. TriNetX’s robust built-in analytics tools facilitated the cohort selection and matching processes. These tools allow for sophisticated event analysis and comparative evaluation of characteristics and outcomes between distinct cohorts. To ensure specificity and accuracy, standardized medical coding systems, such as the International Classification of Diseases, Tenth Revision, Systematized Nomenclature of Medicine, Current Procedural Terminology, and RxNorm codes, were used (9, 10). The requirement for written informed consent was waived, given the reliance of the study on de-identified aggregate data. The Institutional Review Board of Chi Mei Medical Center reviewed and approved this study (11,212-E01).

### Patient selection

The initial study population was defined as individuals aged 40 years and above who received a confirmed diagnosis of COPD between January 1, 2012, and November 01, 2020. To ensure a consistent diagnostic criterion, we excluded individuals diagnosed between January 1, 2010, and December 31, 2011. Eligibility for inclusion required a minimum of three visits to their HCOs and documented evidence of both a pulmonary function test (PFT) and eosinophil count. We divided the eligible population into two groups based on their eosinophil count: the non-eosinophilic group, which included individuals who never had an eosinophil count of 300 cells/μL or above; and the eosinophilic group, comprising those with at least one eosinophil count of 300 cells/μL or above. To minimize confounding variables, we excluded patients with comorbid conditions potentially associated with eosinophilia such as asthma, allergic rhinitis, allergic or atopic dermatitis; those with an unknown allergy history; or those with a history of parasitic infection. The index date for both the study and eosinophilic groups was set as the date of the first COPD diagnosis. The specific codes used for cohort identification are listed in Supplementary Table 1.

### Covariates and outcome measures

In this study, propensity score matching (PSM) was used to align the non-eosinophilic (<300 cells/μL) and eosinophilic groups (≧300 cells/μL) to ensure comparability. Demographic characteristics (age at index, sex, race, and ethnicity); smoking status; comorbid conditions; medication use (including ICS and types of bronchodilators); and baseline pulmonary function, specifically forced expiratory volume (FEV1), as a percentage of the predicted value, were matched. The details of the matching process are provided in Supplementary Table 2.

The primary outcome assessed in this study was the incidence of acute exacerbations during the 3-year follow-up period after the index date. The identification of acute exacerbations of COPD in this study was based on diagnostic coding using the International Classification of Diseases, 10th Revision (ICD-10). This method allowed us to systematically capture clinically recognized exacerbation events as documented in electronic health records.

Secondary outcomes were all-cause mortality and acute respiratory failure (ARF). Additional outcomes of interest included hospitalization rates, emergency department (ED) visits, composite measures of hospitalization or ED visits, systemic glucocorticoid use, and MV requirements. The identification of these outcomes was based on diagnostic and procedural codes, which are delineated in Supplementary Table 3. Two subgroups within the non-eosinophilic group (which included individuals who never had an eosinophil count of 300 cells/μL or above) were identified: (1) patients with eosinophil counts consistently below 100 cells/μL, and (2) patients with eosinophil counts above 100 cells/μl but never exceeding 300 cells/μL (100–299 cell/μL). The codes used to identify these subgroups are displayed in Supplementary Table 4.

### Statistical analysis

The baseline characteristics of the cohort were presented as frequencies and proportions for categorical variables and as mean ± standard deviation for continuous variables. Moreover, PSM was executed using TriNetX built-in tools. These tools facilitated the creation of input matrices based on the selected covariates, followed by logistic regression to calculate the propensity scores for each participant. The patients were matched in a 1:1 ratio using the greedy nearest-neighbour algorithm, with a caliper width of 0.1 pooled standardized difference. The sequence of data entries was randomized to reduce potential bias from the algorithm. The balance between cohorts was evaluated using the standardized difference, with values above 0.1 indicating significant residual imbalance.

Kaplan–Meier survival analysis was used to estimate the probability of various outcomes, including acute exacerbation, survival, hospitalization, ARF, ED visits, composite of hospitalization or ED visits, systemic glucocorticoid use, and MV. The differences in survival curves between the eosinophilic and non-eosinophilic groups were analysed using the log-rank test, and chi-square values were calculated. Cox proportional hazards models were used to estimate hazard ratios (HR) after adjusting for potential confounders. Additionally, a reverse Kaplan–Meier analysis was conducted for non-mortality outcomes using Stata 17.0 and data from the primary Kaplan–Meier analysis. Instances for acute exacerbation, survival, hospitalization, ARF, ED visits, composite of hospitalization or ED visits, systemic glucocorticoid use, and MV between the non-eosinophilic and eosinophilic groups were analysed using the built-in two-sample *t*-test in TriNetX. Additional subgroup analyses with interaction *p*-values for each subgroup were performed using the Wald test. The aforementioned analyses used data from the primary analysis and were conducted using Stata 17.0.

## Results

### Demographic characteristics of included patients

**Figure 1:**
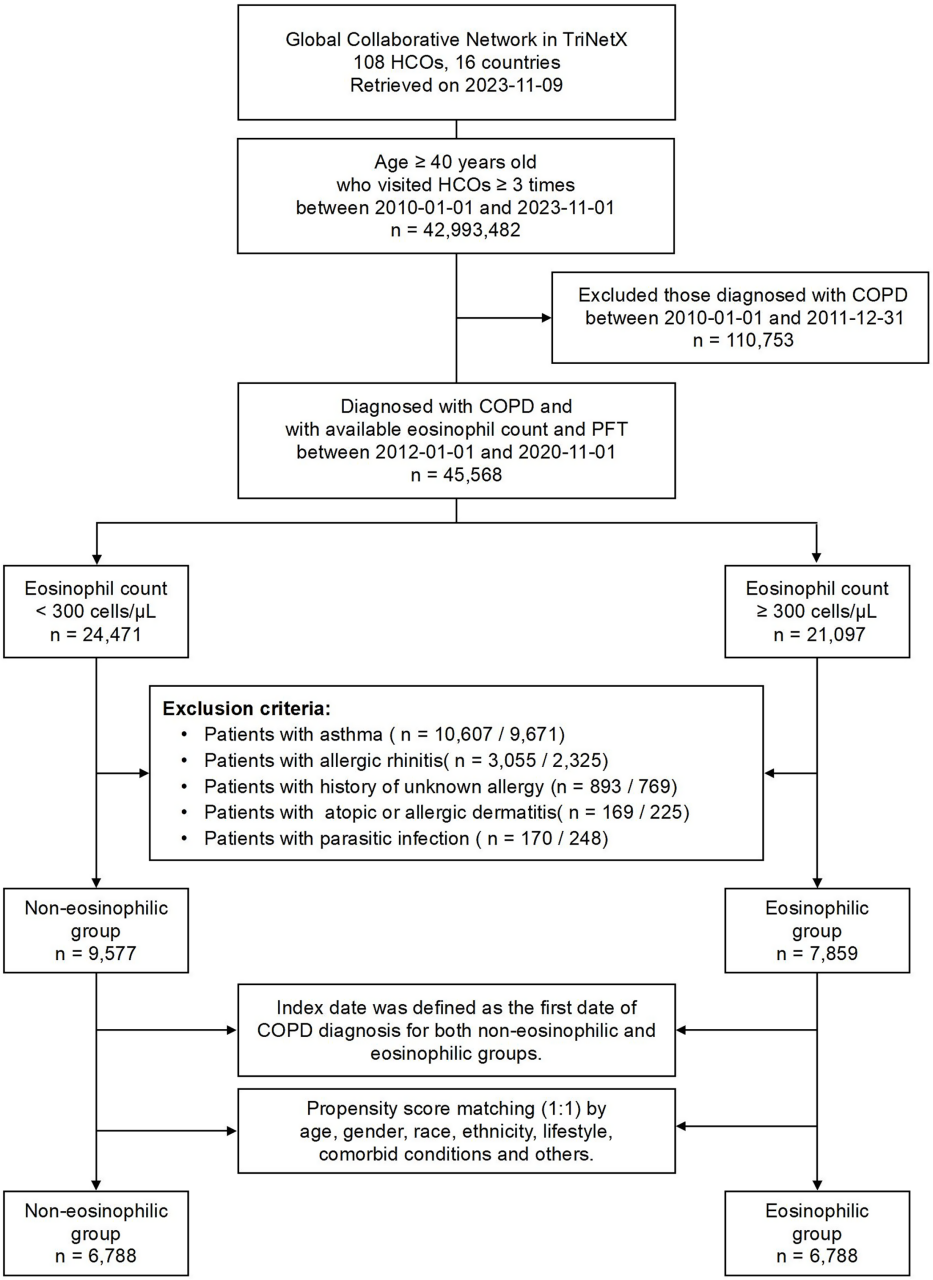
Flowchart of patient selection and cohort construction. HCOs, healthcare organizations; COPD, chronic obstructive pulmonary disease; PFT, pulmonary function test.

As of November 9, 2023, the TriNetX global collaborative network contains data from 108 de-identified HCOs across 16 countries. This dataset included records of 42,993,482 patients aged 40 years or above, who had visited their respective HCOs at least three times between January 1, 2010, and November 1, 2023. A cohort of 45,568 patients was further identified in this study, including those who were diagnosed with COPD between January 1, 2012, and November 1, 2020, and had both eosinophil counts and PFTs available in their records. This group was selected after excluding patients who were diagnosed with COPD between January 1, 2010, and December 31, 2011 (Figure 1).

The eligible population was divided into two groups based on eosinophil count. The division resulted in 24,471 individuals (53.7%) who never had an eosinophil count of 300 cells/μL or above (non-eosinophilic group, <300 cells/μL) and 21,097 individuals (46.3%) who had at least one count reaching or exceeding 300 cells/μL (eosinophilic group, ≧300 cells/μL). After excluding conditions potentially associated with eosinophilia, 9,577 and 7,859 patients were identified in the non-eosinophilic and eosinophilic groups, respectively.

Before matching, notable standardized differences (> 0.1) were observed in several demographic and clinical characteristics. Compared to the eosinophilic group, the non-eosinophilic group had a higher prevalence of women, fewer individuals of unknown race, and more non-Hispanic and Latino individuals. Additionally, the non-eosinophilic group had a significant history of nicotine dependence. Significant differences in comorbidities were also evident at baseline. The eosinophilic group had high incidence of hypertension and chronic kidney disease stages 3 and 4, whereas end-stage renal disease was prevalent in the non-eosinophilic group. Other conditions such as ischemic heart disease, heart failure, diabetes mellitus, pulmonary heart disease, overweight and obesity, past acute exacerbations of COPD, cardiovascular diseases, liver diseases, and immune diseases were more common in the eosinophilic group compared to the non-eosinophilic group. Lastly, the eosinophilic group also exhibited frequent use of bronchodilators and had a high proportion of patients with low haemoglobin counts (< 8 g/dL).

After PSM, the two cohorts were successfully balanced, each comprising 6,788 patients. The matching process effectively minimized baseline differences, achieving standardized differences of less than 0.1 for all matched covariates, as detailed in Table 1. The patients’ age at index ranged from 40 to over 90 years, with a mean of approximately 67 years (SD of approximately 11 years) in both groups. Sex distribution was balanced post-matching, with 56.0% male and 40.6% female in the non-eosinophilic group, and similar proportions were observed in the eosinophilic group. Regarding smoking status, approximately 46.3% were ex-smokers and 36.6% were current smokers in the non-eosinophilic cohort. These variables, along with comorbidities, medication use (e.g., inhaled corticosteroids, bronchodilators), and baseline pulmonary function (FEV1), were included in the PSM process to reduce potential confounding.

**Table 1:** Baseline characteristics in the non-eosinophilic and eosinophilic groups.

	Before matching, No. (%)			After matching, No. (%)		
	Non-eosinophilic group (*n* = 9,577)	Eosinophilic group (*n* = 7,859)	Std diff.	Non-eosinophilic group (*n* = 6,788)	Eosinophilic group (*n* = 6,788)	Std diff.
Demographics						
Age at Index, mean (SD), y	66.3 (10.8)	67.4 (10.9)	0.096	67.2 (10.7)	67 (11.0)	0.002
Sex						
Male	4,933 (51.5)	4,485 (57.1)	0.112	3,800 (56.0)	3,797 (55.9)	0.001
Female	4,411 (46.1)	3,012 (38.3)	0.157	2,756 (40.6)	2,768 (40.8)	0.004
Race						
White	7,954 (83.1)	6,358 (80.9)	0.056	5,606 (82.6)	5,605 (82.6)	<0.001
Black or African American	1,001 (10.5)	641 (8.2)	0.079	596 (8.8)	595 (8.8)	0.001
Asian	51 (0.5)	74 (0.9)	0.048	50 (0.7)	50 (0.7)	<0.001
American Indian or Alaska Native	27 (0.3)	29 (0.4)	0.015	21 (0.3)	24 (0.4)	0.008
Native Hawaiian or Other Pacific Islander	10 (0.1)	10 (0.1)	0.007	10 (0.1)	10 (0.1)	<0.001
Other Race	60 (0.6)	69 (0.9)	0.029	54 (0.8)	53 (0.8)	0.002
Unknown Race	483 (5.0)	684 (8.7)	0.145	460 (6.8)	458 (6.7)	0.001
Ethnicity						
Not Hispanic or Latino	8,873 (92.6)	7,012 (89.2)	0.120	6,173 (90.9)	6,186 (91.1)	0.007
Hispanic or Latino	248 (2.6)	241 (3.1)	0.029	192 (2.8)	194 (2.9)	0.002
Unknown Ethnicity	456 (4.8)	606 (7.7)	0.122	423 (6.2)	408 (6.0)	0.009
Lifestyle (%)						
Ex-smokers	3,882 (40.5)	3,905 (49.7)	0.185	3,140 (46.3)	3,126 (46.1)	0.004
Current smokers	3,665 (38.3)	2,836 (36.1)	0.045	2,484 (36.6)	2,506 (36.9)	0.007
Lower socioeconomic status	710 (7.4)	627 (8.0)	0.021	517 (7.6)	517 (7.6)	<0.001
Body mass index, mean (SD)	28.5 (7.2)	28.9 (7.1)	0.055	28.7 (7.1)	28.9 (7.1)	0.030
Comorbid conditions (%)						
Hypertensive diseases	5,664 (59.1)	5,496 (69.9)	0.227	4,545 (67.0)	4,530 (66.7)	0.005
Chronic kidney disease (CKD)						
CKD, stage 3	799 (8.3)	1,069 (13.6)	0.168	688 (10.1)	715 (10.5)	0.013
CKD, stage 4	216 (2.3)	344 (4.4)	0.118	191 (2.8)	208 (3.1)	0.015
CKD stage 5	46 (0.5)	106 (1.3)	0.091	44 (0.6)	53 (0.8)	0.016
End stage renal disease	722 (7.5)	359 (4.6)	0.125	279 (4.1)	312 (4.6)	0.024
Ischemic heart diseases	3,343 (34.9)	3,409 (43.4)	0.174	2,764 (40.7)	2,761 (40.7)	0.001
Heart failure	2,425 (25.3)	2,663 (33.9)	0.188	2,076 (30.6)	2,056 (30.3)	0.006
Diabetes mellitus	2,401 (25.1)	2,639 (33.6)	0.188	2,061 (30.4)	2,062 (30.4)	<0.001
Sleep apnoea	1,864 (19.5)	1,816 (23.1)	0.089	1,500 (22.1)	1,472 (21.7)	0.010
Pulmonary heart disease	1,724 (18.0)	1,816 (23.1)	0.127	1,417 (20.9)	1,410 (20.8)	0.003
Overweight and obesity	1,724 (18.0)	1,839 (23.4)	0.134	1,437 (21.2)	1,421 (20.9)	0.006
AECOPD	1,553 (16.2)	1,578 (20.1)	0.100	1,263 (18.6)	1,258 (18.5)	0.002
Cerebrovascular diseases	1,434 (15.0)	1,505 (19.2)	0.111	1,190 (17.5)	1,183 (17.4)	0.003
Liver diseases	1,092 (11.4)	1,123 (14.3)	0.086	894 (13.2)	879 (12.9)	0.007
Lung cancer	918 (9.6)	940 (12.0)	0.077	765 (11.3)	743 (10.9)	0.010
Immune disorders	380 (4.0)	548 (7.0)	0.132	349 (5.1)	346 (5.1)	0.002
Psychiatric disorders						
Mood disorders	2,194 (22.9)	2,120 (27.0)	0.094	1,692 (24.9)	1,717 (25.3)	0.008
Schizophrenia	256 (2.7)	300 (3.8)	0.065	214 (3.2)	234 (3.4)	0.016
Laboratory (%)						
Haemoglobin level < 8 g/dL	671 (7.0)	1,099 (14.0)	0.229	648 (9.5)	680 (10.0)	0.016
FEV1/Predicted, mean (SD), %	64.3 (20.9)	64.8 (21.0)	0.021	64.2 (21.2)	64.9 (21.2)	0.030

AECOPD, acute exacerbations of chronic obstructive pulmonary disease; CKD, chronic kidney disease; FEV1, forced expiratory volume in 1 s; PSM, propensity score matching; SD, standard deviation; Std diff., standardized difference.

### Primary outcome

**Figure 2:**
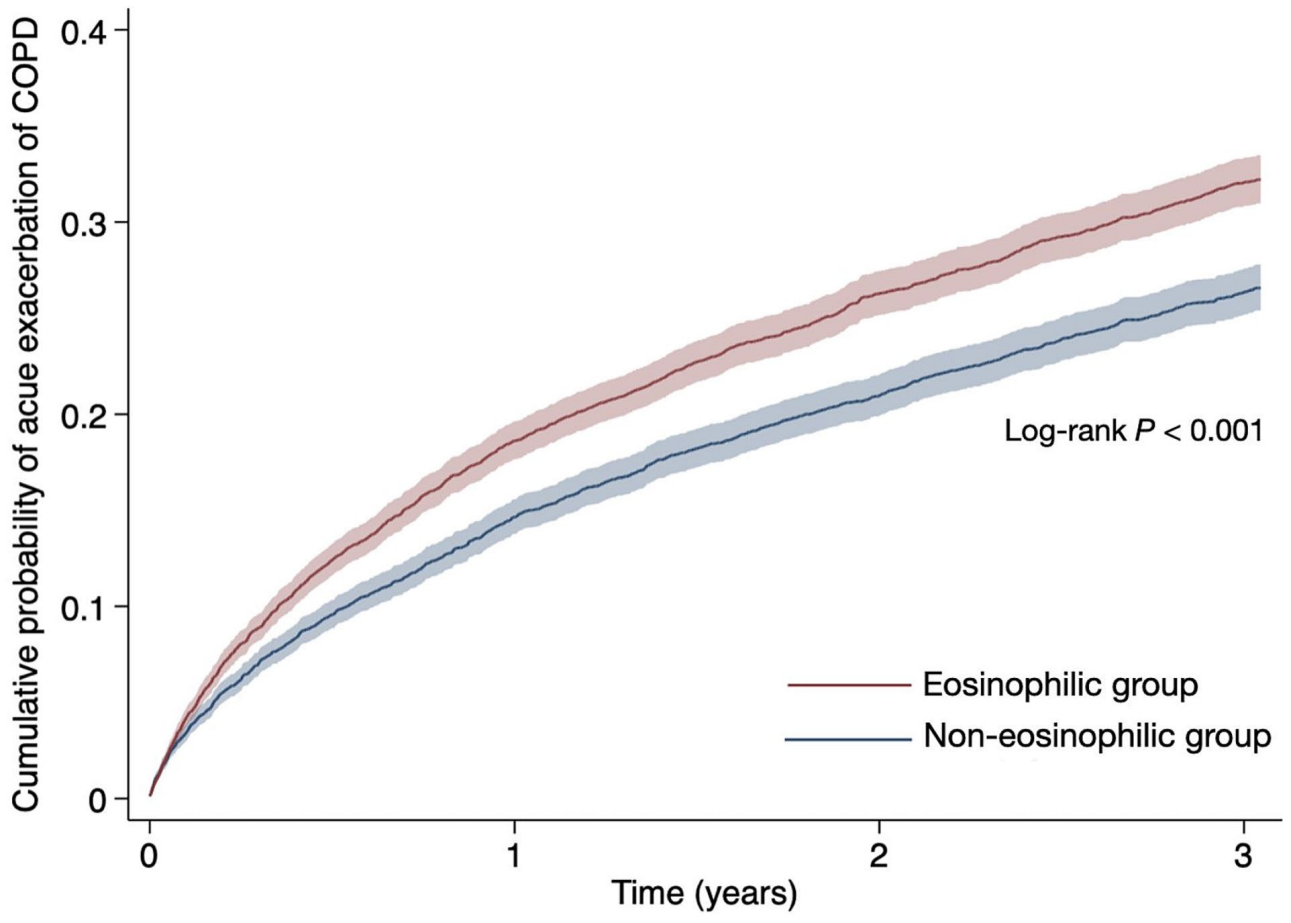
Cumulative probability of acute exacerbation of chronic obstructive pulmonary diseases during the three-year follow-up period.

During the 3-year follow-up, the non-eosinophilic group (<300 cells/μL) experienced fewer acute exacerbations of COPD than the eosinophilic group (≧300 cells/μL), with 1,435 (21.1%) and 1,787 (26.3%) affected individuals, respectively. The Cox proportional hazard model analysis revealed a significantly low incidence of acute exacerbation in the non-eosinophilic group, corresponding to a 1.27-fold lower risk compared to the eosinophilic group (HR, 0.790; 95% CI, 0.74–0.85), as detailed in Table 2. Kaplan–Meier survival analysis further corroborated these findings, demonstrating a higher event-free rate for the non-eosinophilic group (73.4%) than that for the eosinophilic group (67.8%) by the end of the follow-up period, with the difference being statistically significant (log-rank test, *p* < 0.001, Figure 2).

**Table 2:** The hazard ratio and incidence for comparing matched non-eosinophilic and eosinophilic COPD groups for each outcome.

Outcomes	Non-eosinophilic group event no. (%)	Eosinophilic group event no. (%)	HR (95%CI)	*P*-value
Primary outcomes				
Acute exacerbation	1,435 (21.1)	1,787 (26.3)	0.790 (0.737,0.846)	< 0.001
Secondary outcomes				
All-cause mortality	1,264 (18.6)	1,561 (23.0)	0.818 (0.759,0.881)	< 0.001
Acute respiratory failure	990 (14.6)	1,357 (20.0)	0.721 (0.664,0.783)	< 0.001
Other outcomes				
Hospitalization	2,713 (40.0)	3,047 (44.9)	0.870 (0.827,0.917)	< 0.001
ED visit	2,419 (35.6)	2,749 (40.5)	0.853 (0.807,0.901)	< 0.001
Hospitalization or ED visit	3,611 (53.2)	3,981 (58.6)	0.867 (0.829,0.907)	< 0.001
Systemic glucocorticoid use	2,497 (36.8)	2,724 (40.1)	0.908 (0.860,0.959)	< 0.001
Mechanical ventilation	656 (9.7)	905 (13.3)	0.723 (0.654,0.800)	< 0.001

CI, confidence interval; HR, hazard ratio; ED, emergency department.

Instance-level analysis that measured the mean number of acute exacerbations reported a mean of 3.5 ± 4.8 events in the non-eosinophilic group, as opposed to 3.8 ± 5.9 events in the eosinophilic group. Despite the low mean number of events observed in the non-eosinophilic group, this difference was not statistically significant, with a *t*-value of −1.43 and a *p*-value of 0.15 (Supplementary Table 5). Therefore, while the overall risk of exacerbation was reduced in the non-eosinophilic group, the results did not demonstrate a significant difference in the number of exacerbations experienced by individuals between the groups.

### Secondary outcomes

**Figure 3:**
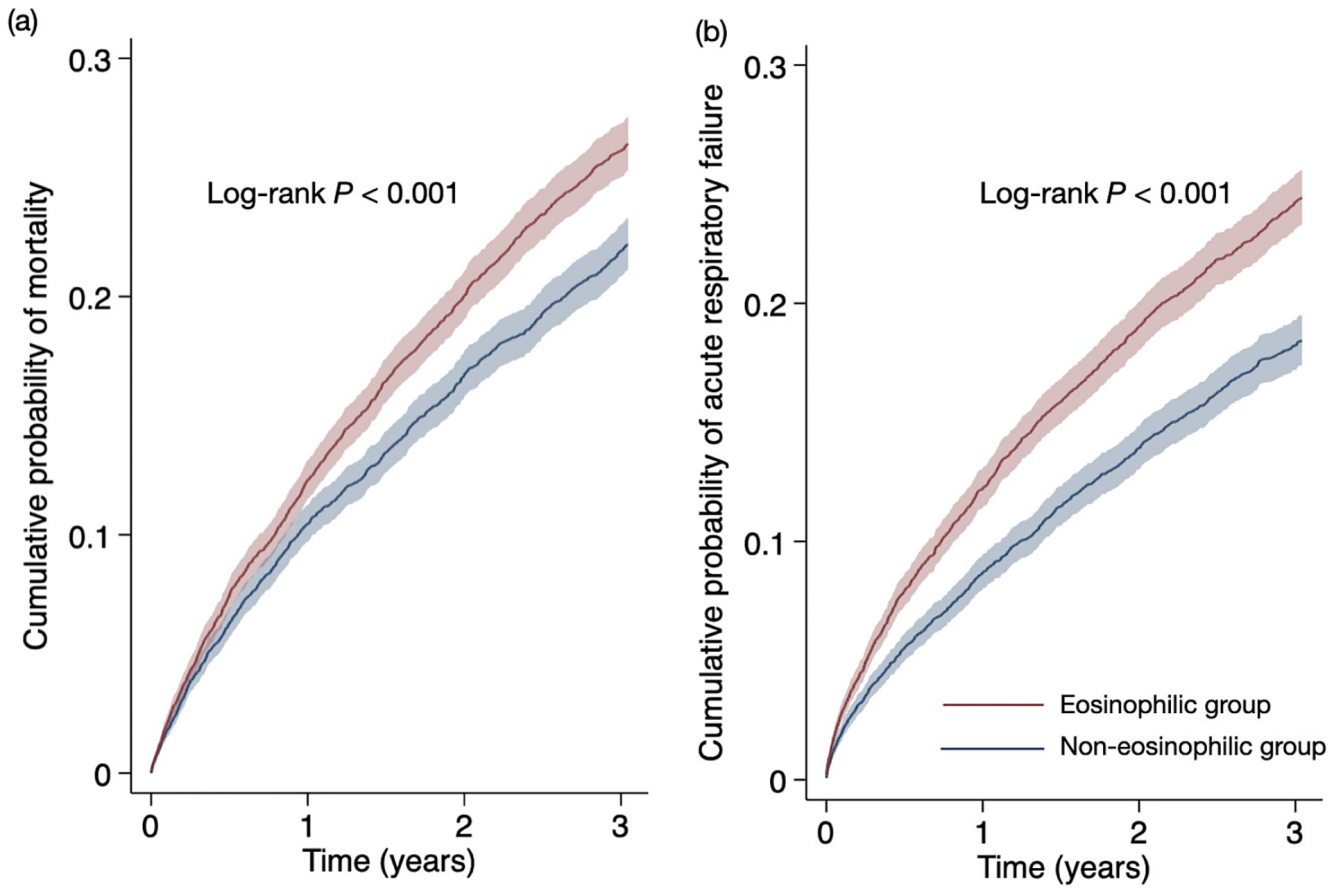
Cumulative probability of **(a)** all-cause mortality and **(b)** acute respiratory during the three-year follow-up period.

In this study, the secondary outcomes assessed were all-cause mortality and risk of ARF over the prespecified 3-year follow-up period. The observations indicated 1,264 and 1,561 deaths in the non-eosinophilic and eosinophilic groups, respectively. The Cox proportional hazard model analysis displayed a significantly lower mortality rate in the non-eosinophilic group compared to the eosinophilic group (18.6% vs. 23.0%; HR, 0.818; 95% CI, 0.76–0.88), as indicated in Table 2. Kaplan–Meier survival analysis further revealed a higher survival rate in the non-eosinophilic group (77.8%) than that in the eosinophilic group (73.6%) after follow-up (log-rank test, *p* < 0.001, Figure 3a).

Concerning ARF, the non-eosinophilic group exhibited a reduced risk (14.6% vs. 20.0%; HR, 0.721; 95% CI, 0.66–0.78), detailed in Table 2. Kaplan–Meier analysis demonstrated a lower incidence of ARF in the non-eosinophilic group, with an event-free probability of 81.6%, compared to 75.6% in the eosinophilic group (log-rank test, *p* < 0.001, Figure 3b). The instance-level analysis concerning the mean number of ARF events reported an average of 2.8 ± 3.7 events in the non-eosinophilic group, in contrast to 3.2 ± 4.9 events in the eosinophilic group, where the difference was statistically significant (*t*-value = −2.52; *p* = 0.012).

### Other outcomes

Statistically significant differences in risk were noted between the groups for several outcomes, including hospitalization rates, a composite measure of hospitalization or ED visits, systemic glucocorticoid use, and the need for MV, with log-rank *p*-values less than 0.001, signifying a uniform trend of reduced risk for patients with eosinophil counts under 300 cells/μL, as presented in Table 2 and Supplementary Figures 1a–e. Hospitalization was recorded in 40.0% of the non-eosinophilic group and 44.9% of the eosinophilic group, with HR of 0.870 (95% CI, 0.827–0.917). For ED visits, a lower percentage was reported in the non-eosinophilic group (35.6%) in contrast to the eosinophilic group (40.5%; HR, 0.853; 95% CI, 0.807–0.901). The non-eosinophilic group also had fewer combined hospitalizations or ED visit events (53.2%) than the eosinophilic group (58.6%; HR, 0.867; 95% CI, 0.829–0.907). The rate of systemic glucocorticoid use was reduced in the non-eosinophilic group (36.8%) than that in the eosinophilic group (40.1%; HR, 0.908; 95% CI, 0.860–0.959), and the need for MV was less frequently observed in the non-eosinophilic group (9.7% vs. 13.3%; HR, 0.723; 95% CI, 0.654–0.800). In instance-level analysis, only the difference in ED visits was significant (Supplementary Table 5).

### Subgroup analysis

**Figure 4:**
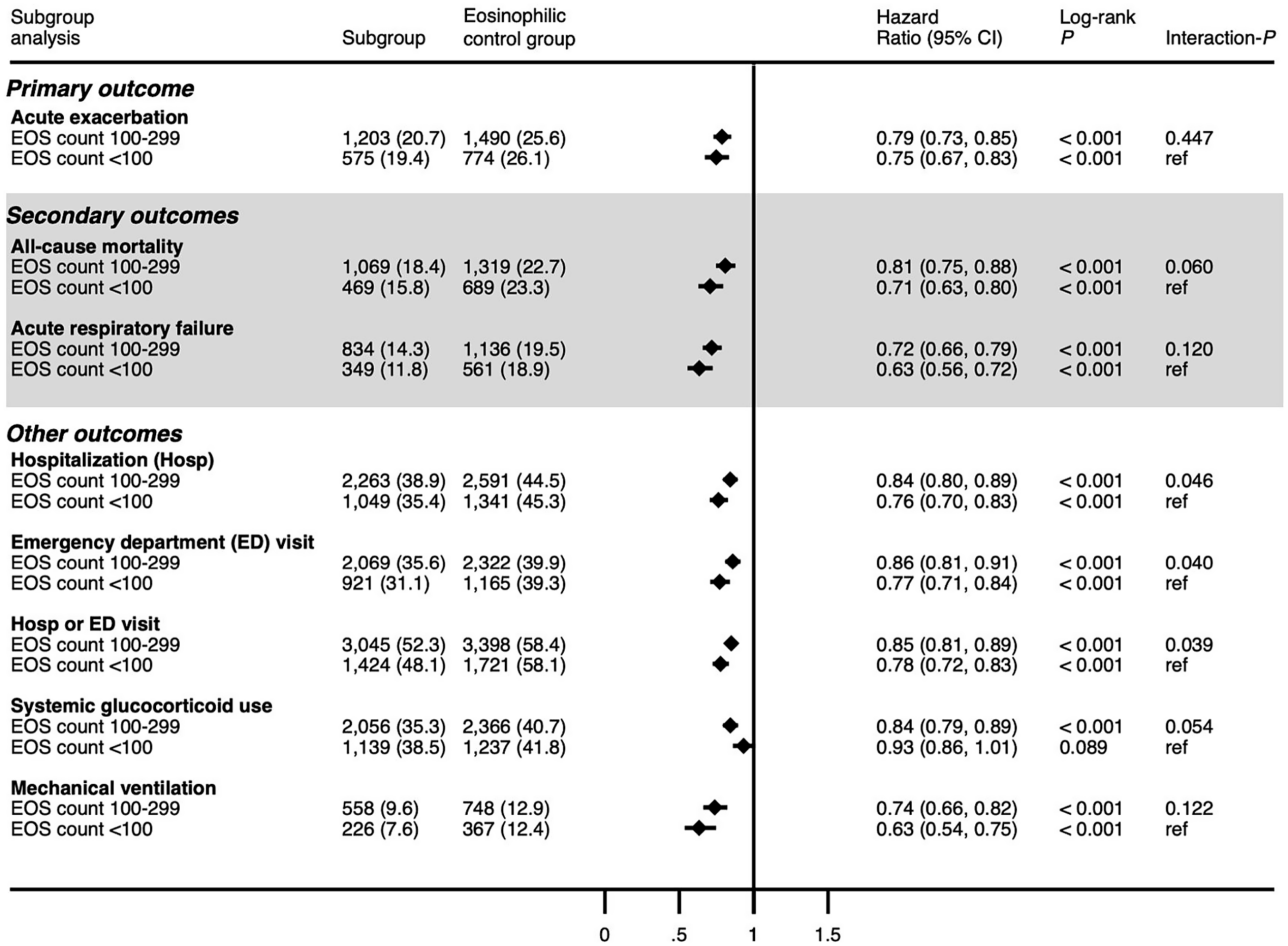
Hazard ratio of outcomes in the non-eosinophilic subgroups compared with the eosinophilic group. EOS, eosinophil.

Figure 4 displays the HRs for the two defined subgroups within the study cohort relative to the eosinophilic group, with each subgroup distinguished by the eosinophil count thresholds. The first subgroup consisted of individuals with eosinophil counts that were consistently below 100 cells/μL. The second subgroup was composed of individuals with eosinophil counts above 100 cells/μL but never exceeding 300 cells/μL. Cox proportional hazard model analyses demonstrated that statistically significant reductions in risk were consistent across all outcomes for the aforementioned subgroups compared with the eosinophilic group. Specifically, the interaction *p*-values (int-*P*) highlighted a potential correlation between low eosinophil counts and decreased risks of hospitalization (int-*p* = 0.046), ED visits (int-*p* = 0.040), and the combined outcome of hospitalization and ED visits (int-*p* = 0.039). For the other assessed outcomes, the differences between the subgroups were not statistically significant. A direct comparison between the two non-eosinophilic subgroups was also conducted and is presented in Supplementary Table 6. Statistically significant associations were observed, showing lower risks of ED visits (HR, 0.909; 95% CI, 0.831–0.994; *p* = 0.037) and the combined outcome of hospitalization and ED visits (HR, 0.926; 95% CI, 0.861–0.996; *p* = 0.040) in patients with lower eosinophil counts (<100 cells/μL) compared to those with slightly higher counts (100–299 cells/μL).

Lastly, an additional subgroup analysis based on inhaler usage patterns was conducted. The triple inhaler group, comprising a long-acting beta-agonist (LABA), a long-acting muscarinic antagonist (LAMA), and an inhaled corticosteroid (ICS), demonstrated a significantly lower risk of acute exacerbation in the eosinophilic group compared to the non-eosinophilic group (HR, 0.86; 95% CI, 0.80–0.93), as shown in Supplementary Table 7. No statistically significant differences were observed in the other inhaler subgroups. Furthermore, both the LABA plus ICS group and the triple inhaler group were associated with significantly reduced risks of all-cause mortality and acute respiratory failure in the eosinophilic group, as presented in Supplementary Table 8.

## Discussion

The nationwide survey for predicting the risk of acute exacerbation, all-cause mortality, and acute respiratory failure (ARF) in COPD using blood eosinophil counts, with a cut-off value of 300 cells/μL, has not been conducted. Our studies demonstrated that blood eosinophil counts at 300 cells/μL or higher were associated with a significant risk of acute exacerbation, respiratory failure, and all-cause mortality, and this information offers practical and clinically relevant distinctions that have the potential to guide management strategies.

Furthermore, COPD exacerbations vary in terms of inflammation and causative factors. Eosinophils play a complex role in COPD with acute exacerbations. Although the involvement of eosinophils has been associated with exacerbation in some studies, this relationship remains controversial. Exacerbations are commonly associated with increased neutrophilicity and, to a lesser degree, eosinophilic airway inflammation (11). Respiratory viral and bacterial infections are frequently implicated as primary causes of exacerbation (4, 12–14). A study by Bafadhel et al. (15) reported that the causes of exacerbation events were as follows: bacterial infection alone (37%), sputum eosinophilia alone (17%), bacterial and viral co-infection (12%), bacterial infection with sputum eosinophilia (6%), and viral infection with sputum eosinophilia (3%).

Other studies have also confirmed the presence of eosinophils in both large and small airway tissue samples, as well as in 20–40% of induced sputum samples from patients with stable COPD. Moreover, airway eosinophilia tends to increase during exacerbations (16). Eosinophils play an important role in COPD exacerbation, and the authors discovered that the peripheral eosinophil percentage can be a responsive indicator of sputum eosinophilia.

Our findings are consistent with those of previous studies. In one study examining two multicentre longitudinal cohorts, patients with moderate-to-severe COPD exhibited a linear increase in exacerbation risk, corresponding to high blood eosinophil counts. These findings underscore the heightened risk of COPD exacerbation associated with elevated eosinophil counts. They also established a threshold blood eosinophil count of ≥300 cells/μL as indicative of exacerbations, validating this cutoff as a predictor for future exacerbations through prospective data from the Evaluation of COPD Longitudinally to Identify Predictive Surrogate End-points (ECLIPSE) study (17).

In a prior investigation within the ECLIPSE study, the aim was to establish the prevalence of COPD in participants with persistently elevated eosinophil levels (≥2%) in both blood and sputum over a 3-year follow-up period. The results revealed 88% concordance between blood eosinophil thresholds of 2% and 150 cells/μL (18). Numerous subsequent studies have utilized a low threshold of 150 cells/μL for eosinophil counts (19–23).

In our investigation, we observed that elevated baseline eosinophil levels were correlated with an increased risk of future exacerbations over 3 years. The absolute eosinophil count was consistently associated with increased exacerbation rates. The relevance of the absolute eosinophil count may be biologically significant considering the variability in total white blood cell counts, and a single percentage value may not encompass the entire spectrum of blood eosinophils. Our findings are similar to those of previous studies. Vedel-Krogh et al. (24) reported that patients with COPD with blood eosinophil levels > 340 cells/L exhibited a 1.76-fold increased risk of severe exacerbations after a median follow-up period of 3.3 years. Zeiger et al. (25) reported that high blood eosinophil counts (≥300 cells/mm3) were an independent risk factor for future exacerbations in COPD. Similar to our investigation, these investigations included sizable cohorts of individuals with moderate-to-severe COPD (FEV1 < 80%) and adopted elevated eosinophil cutoffs based on absolute counts (≥300 cells/mm3), rather than relying solely on percentage cutoffs for eosinophils. Studies that reported a lack of association between blood eosinophils and COPD exacerbations differed from our study, with many of them having small sample populations (26–29).

### Mortality

Prior studies have identified an association between elevated blood eosinophil concentrations and reduced in-hospital mortality as well as a short length of stay in critically ill patients experiencing acute exacerbations of COPD (17). Holland et al. (30) enrolled 65 patients with COPD and discovered that eosinophil count might be a useful marker of severity in patients with COPD with acute exacerbation. Additionally, they also established that mortality was low in the eosinopenia group. Another study (31) enrolled 44 patients with COPD with eosinopenia (eosinophil count < 40/mm^3^ on the day of admission) and 56 patients without eosinopenia. The eosinopenia group had increased in-hospital mortality, higher risk of MV, and longer duration of hospitalization than those without eosinopenia. Another retrospective study (32) enrolled 647 patients with exacerbations and ARF who were admitted to the intensive care unit (ICU). They identified that patients with COPD with peripheral eosinophil levels of >2% had improved outcomes. One study (33) included 110 patients in the analysis, using eosinophil cutoff points of 2% and 150 cells, and identified increased mortality in those with <2% eosinophils and <150 cells. Studies reporting an opposing association between blood eosinophil count and mortality diverged from our investigation, primarily because many of them had small sample sizes and employed percentage cutoff values for eosinophils. In contrast, one study revealed that individuals with both eosinophilia and a history of asthma faced an elevated risk of mortality from COPD compared to those without eosinophilia or a history of asthma. The presence of “asthma attacks with eosinophilia” might indicate an increased risk of mortality from COPD, adding to the well-established risk factors for mortality in COPD. Given that unstable or clinically active airway inflammation is associated with sputum and peripheral blood eosinophilia, it is conceivable that more severe symptoms and overall disease severity may contribute to extensive airway remodeling. Although speculative, this could potentially lead to irreversible airway obstruction, progression of COPD, and ultimately mortality from COPD (34).

Our study included a large COPD population, utilized a 300 cells/μL eosinophil cut-off, and observed a discrepancy in mortality linked to blood eosinophil counts. No apparent distinctions were identified in the baseline demographic characteristics, suggesting that this observation may be indicative of the COPD phenotype or other underlying patient traits. The reason why elevated eosinophil levels in COPD may predict poor prognosis remains uncertain. Further research is required to establish the association between eosinophil count and COPD mortality, and additional investigations are warranted to determine the optimal cutoff value.

### Acute respiratory failure

Limited information is available on eosinophils and ARF in patients with COPD. Yu et el (35). demonstrated that no differences were observed among the eosinophil ≤ 100 cells/μL, 100 < eosinophil < 300 cells/μL, and eosinophil ≥ 300 cells/μL regarding the admission rate to the ICU, MV, and in-hospital mortality. Cui et al. (36) enrolled 650 patients with COPD after PSM and detected no significant difference between eosinophil counts (<2% and ≧2%) and respiratory failure. In our study, we discovered that eosinophil count increased the risk of ARF. Several possible explanations exist for this observation. In a restricted group of patients with moderate-to-severe COPD, a correlation was observed between reduced lung function values and elevated sputum concentrations of both eosinophils and eosinophil cationic proteins. This suggests a potential link between airway eosinophilia and airflow obstruction (37).

A previous study revealed that markers indicative of T cell and eosinophilic inflammation act as predictors of COPD progression. Patients with COPD with stable disease display elevated plasma levels of interleukin-2, in contrast to those with rapidly progressive COPD and lung function decline. Additionally, they exhibit lower plasma eotaxin-1 levels compared to the normal population. These findings imply that cell-mediated immune responses significantly affect the COPD disease status (38). Furthermore, elevated eosinophil levels are associated with acute COPD exacerbation, and frequent exacerbations lead to lung function impairment and disease progression, ultimately resulting in ARF.

Although eosinophils are associated with inflammatory and autoimmune diseases, the exact clinical significance and pathophysiology of elevated eosinophil counts in COPD remain debatable. Given the complexity of COPD, it is conceivable that eosinophil count may be specifically related to ARF within certain patient subgroups. For future studies taking into account other factors associated with the development of ARF is crucial. The strength of this study is the inclusion of a multiple-country database and large cohorts of well-characterized patients with COPD excluding patients with asthma, asthma-COPD overlap, and allergic disease. We identified an eosinophil threshold that increased exacerbation risk, all-cause mortality, ARF, systemic steroid use, and MV.

However, some studies conducted within observational cohorts have not consistently demonstrated a clear or reliable association between peripheral blood eosinophil levels and clinical outcomes or treatment responses in patients with COPD. The variability in study designs, populations, and definitions of eosinophilic thresholds may contribute to these inconsistent findings. Interestingly, one study conducted in a general U.S. population found that peripheral blood eosinophil levels greater than 2% were associated with a lower burden of comorbidities, suggesting a potentially protective or distinct biological profile (39). In a cohort study involving Japanese patients with COPD, approximately 19% of individuals were found to have peripheral blood eosinophil counts exceeding 300 cells/mm^3^. Notably, the presence of eosinophilia in this population was not associated with an increased risk of COPD exacerbations, suggesting that elevated eosinophil levels did not correspond to worsened short-term clinical outcomes. However, the study did reveal a significant association between higher eosinophil counts and a reduced risk of all-cause mortality (40). In another observational cohort study, researchers examined the relationship between peripheral blood eosinophil levels and various clinical outcomes by stratifying patients based on different eosinophil thresholds, specifically 2, 3, and 4%. The analysis revealed no significant differences in the rates of COPD exacerbations, history of asthma, or three-year survival among individuals with eosinophil counts above or below these thresholds. These findings suggest that varying levels of blood eosinophils, within this range, may not serve as reliable predictors of disease severity, comorbid conditions, or long-term outcomes in the studied population (28). In a prospective cohort study conducted in Korea, authors reported that patients with elevated peripheral blood eosinophil levels experienced several favorable clinical outcomes compared to those with lower eosinophil counts. Specifically, individuals with higher eosinophil levels demonstrated significantly higher overall survival rates. Additionally, these patients reported fewer and less severe respiratory symptoms, along with notable improvements in health-related quality of life measures. These findings suggest that elevated eosinophil levels may be associated with a more favorable disease trajectory in certain subgroups of patients with COPD (41). The relationship between blood eosinophil levels and the risk of exacerbations in patients with COPD remains a subject of ongoing debate. While some studies have suggested a potential link between elevated eosinophil counts and increased exacerbation risk or treatment responsiveness, others have failed to demonstrate consistent or clinically meaningful associations. This inconsistency underscores the continued uncertainty surrounding the use of eosinophil levels as a reliable biomarker for guiding COPD management and predicting patient prognosis. Further research is needed to clarify the conditions under which eosinophil measurements may offer actionable insights and to establish standardized thresholds that can be applied across diverse patient populations.

### Limitation

The following limitations should be acknowledged. First, although blood eosinophil counts were measured, no corresponding data on sputum eosinophil counts was present. Additionally, the timing of the eosinophil count was inconsistent across patients. There exists a potential for variability in eosinophil counts depending on the disease state, particularly during periods of clinical stability versus acute exacerbations. One of the limitations is the lack of clarity regarding whether the eosinophil measurements in current studies were obtained during acute hospital admissions or when patients were in a stable condition. This ambiguity complicates the interpretation of findings and limits the ability to draw definitive conclusions about the role of eosinophils in different phases of COPD. We emphasize the importance of future research efforts that carefully document and standardize the timing of eosinophil count assessments. Additionally, future studies should explore the temporal fluctuations in eosinophil levels throughout the course of COPD exacerbations. Such investigations are essential to gain deeper, more nuanced insights into the clinical relevance of eosinophilic inflammation and its potential role in guiding personalized treatment strategies. Moreover, patients may have received corticosteroid treatment before admission, which may have influenced their eosinophil values. However, we could not eliminate these variables because they introduced a potential source of bias. Although a dedicated COPD group was not available in our study, we addressed disease severity by employing a matching strategy based on patients’ prior history of COPD exacerbations. In addition, we incorporated PFT results as surrogate markers to further account for the variability in disease severity. These methodological approaches allowed us to approximate the impact of COPD status and ensure a more balanced comparison between study groups, despite the absence of a formally defined COPD cohort. In this study, we included only patients with three or more healthcare encounters to ensure the availability of sufficient clinical data. However, we did not adjust for the number of healthcare visits between groups, which may have influenced the likelihood of detecting elevated eosinophil counts. The probability of observing more than one blood eosinophil count greater than 300 cells/μL may increase with more frequent healthcare visits. This factor could partially explain the observed associations with exacerbations and comorbidities. Other limitations include the absence of detailed patient-level information, which restricted our ability to analyze specific clinical variables and individual characteristics. Additionally, reliance on administrative coding data introduces the possibility of coding inaccuracies or misclassifications. The retrospective design further limits causal inference and may be associated with potential selection bias. Moreover, inherent constraints related to the data source may limit the generalizability of our findings to broader or more diverse patient populations.

## Data Availability

The raw data supporting the conclusions of this article will be made available by the authors, without undue reservation.
