# Supplementary figures and images for: Association between eosinophil count and prognosis in chronic obstructive pulmonary disease patients
Source: Front Med (Lausanne). 2025 Sep 3;12:1525709. doi: 10.3389/fmed.2025.1525709 (PMC12440774; doi:10.3389/fmed.2025.1525709)

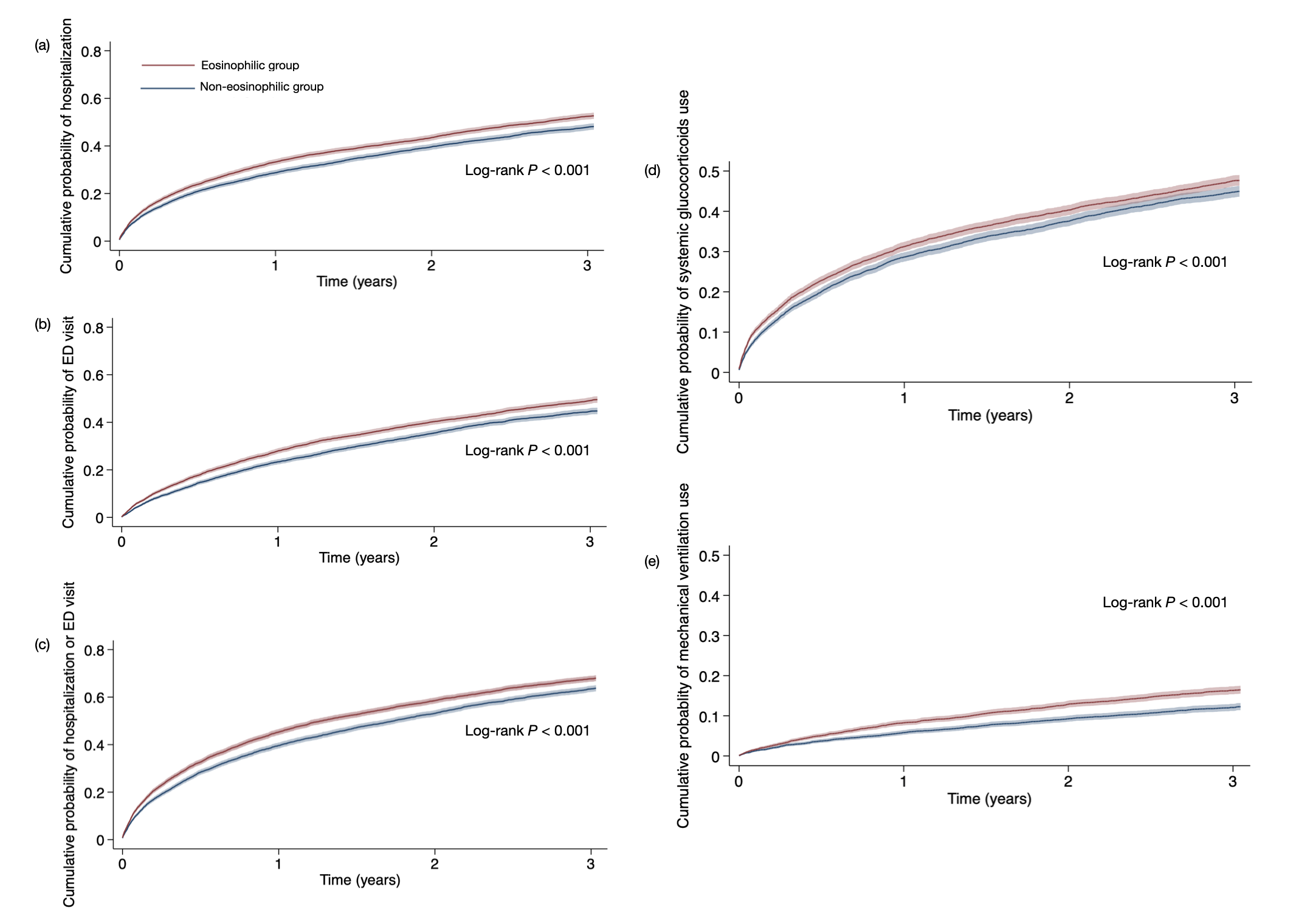

Supplement: Supplementary file 1 [file Supplementary_file_1.docx]
