# Supplementary material for: Association between eosinophil count and prognosis in chronic obstructive pulmonary disease patients
Source: Front Med (Lausanne). 2025 Sep 3;12:1525709. doi: 10.3389/fmed.2025.1525709 (PMC12440774; doi:10.3389/fmed.2025.1525709)
Supplement: Supplementary file 2 [file Table_1.docx]

| **Supplementary Table 1. Demographic, diagnostic, procedural, medication, visit, and laboratory codes utilized in the definition of the cohorts.** | | |
| --- | --- | --- |
| **Category** | **Code** | **Description** |
| **#1 Age at least 40 years** | |  |
| demographics | Age | Age (at least 40 years) |
| **#2 Visited HCOs at least 3 times between Jan 1, 2010 and Nov 1, 2023** | | |
| (must have any of the following between Jan 1, 2010 and Nov 1, 2023) | | |
| visit | TNX:Visit | Number of occurrences: greater than or equal to 3 instances |
| **#3 Excluded those diagnosed with COPD between Jan 1, 2010 and Dec 31, 2011** | | |
| (must not have the following between Jan 1, 2010 and Dec 31, 2011) | | |
| diagnosis | UMLS:ICD10CM:J44 | Other chronic obstructive pulmonary disease |
| **#4 Diagnosed with COPD and with pulmonary function test (PFT) available between Jan 1, 2012 and Nov 1, 2020** | | |
| (must have the following between Jan 1, 2010 and Dec 31, 2011) | | |
| diagnosis | UMLS:ICD10CM:J44 | Other chronic obstructive pulmonary disease |
| laboratory | TNX:FINDING:2005 | Pulmonary Function Test (PFT) (at least -1024.00 units) |
| **#5 Eosinophlic group: with eosinophil count ever greater than or equal to 300#/volume between Jan 1, 2012 and Nov 1, 2020** | | |
| (must have the following between Jan 1, 2012 and Nov 1, 2020) | | |
| laboratory | TNX:LG32849-8 | Eosinophils [#/volume] in Blood (at least 0.30 10*3/uL) |
| **#6 Non-eosinophlic group: with eosinophil count never greater than or equal to 300#/volume between Jan 1, 2012 and Nov 1, 2020** | | |
| (must not have #5 and must have the following Jan 1, 2012 and Nov 1, 2020) | | |
| laboratory | TNX:LG32849-8 | Eosinophils [#/volume] in Blood (between 0.00 and 0.30 10*3/uL) |
| **#7 Excluded diseases associated with potential eosinophilia** | | |
| (must not have any of the following comorbid condition occurred within 1 year and 1 month before the diagnosis using #3) | | |
| diagnosis | UMLS:ICD10CM:J45 | Asthma |
| diagnosis | UMLS:ICD10CM:J30 | Vasomotor and allergic rhinitis |
| diagnosis | UMLS:ICD10CM:L20 | Atopic dermatitis |
| diagnosis | UMLS:ICD10CM:L23 | Allergic contact dermatitis |
| diagnosis | UMLS:ICD10CM:Z88.9 | Allergy status to unspecified drugs, medicaments and biological substances |
| diagnosis | UMLS:ICD10CM:B65-B83 | Helminthiases |
| diagnosis | UMLS:ICD10CM:B85-B89 | Pediculosis, acariasis and other infestations |
| diagnosis | UMLS:ICD10CM:B90-B94 | Sequelae of infectious and parasitic diseases |

| **Supplementary Table 2.** Demographic, diagnostic, and laboratory codes utilized in the definition of covariates. | | | |
| --- | --- | --- | --- |
| **Category** | **Code** | **Description** |  |
| demographics | AI | Age at Index |  |
| demographics | F | Female |  |
| demographics | M | Male |  |
| demographics | 56370 | Black or African American |  |
| demographics | 75301 | White |  |
| demographics | 1002-5 | American Indian or Alaska Native |  |
| demographics | 46997 | Asian |  |
| demographics | 64498 | Native Hawaiian or Other Pacific Islander |  |
| demographics | 84373 | Other Race |  |
| demographics | UNK | Unknown Race |  |
| demographics | 104582 | Not Hispanic or Latino |  |
| demographics | 85865 | Hispanic or Latino |  |
| demographics | UN | Unknown Ethnicity |  |
| Diagnosis | E66 | Overweight and obesity |  |
| Diagnosis | I10-I16 | Hypertensive diseases |  |
| Diagnosis | I20-I25 | Ischemic heart diseases |  |
| Diagnosis | I26-I28 | Pulmonary heart disease and diseases of pulmonary circulation |  |
| Diagnosis | I60-I69 | Cerebrovascular diseases |  |
| Diagnosis | K70-K77 | Diseases of liver |  |
| Diagnosis | I50 | Heart failure |  |
| Diagnosis | E08-E13 | Diabetes mellitus |  |
| Diagnosis | C00-D49 | Neoplasms |  |
| Diagnosis | D80-D89 | Certain disorders involving the immune mechanism |  |
| Diagnosis | N18 | Chronic kidney disease (CKD) |  |
| Diagnosis | F17 | Nicotine dependence |  |
| Diagnosis | Z87.891 | Personal history of nicotine dependence |  |
| Diagnosis | Z55-Z65 | Persons with potential health hazards related to socioeconomic and psychosocial circumstances |  |
| Diagnosis | F20-F29 | Schizophrenia, schizotypal, delusional, and other non-mood psychotic disorders |  |
| Diagnosis | F30-F39 | Mood [affective] disorders |  |
| Diagnosis | G47.3 | Sleep apnea |  |
| Diagnosis | G47.33 | Obstructive sleep apnea (adult) (pediatric) |  |
| Diagnosis | C34 | Malignant neoplasm of bronchus and lung |  |
| Laboratory | 20152-5 | FEV1/Predicted |  |
| Laboratory | 9014 | Hemoglobin [Mass/volume] in Blood |  |

| **Supplementary Table 3.** Diagnostic, visit, and procedural codes utilized in the definition of outcomes. | | |
| --- | --- | --- |
| Index date: the first date of diagnosis of COPD for both eosinophilic and non-eosinophilic group | | |
| Outcome time window: 1 month to 3 years after the index date | |  |
| **Category** | **Code** | **Description** |
| **#1 Acute exacerbation** | | |
| Diagnosis | UMLS:ICD10CM:J44.1 | Chronic obstructive pulmonary disease with (acute) exacerbation |
| Diagnosis | UMLS:ICD10CM:J44.0 | Chronic obstructive pulmonary disease with (acute) lower respiratory infection |
| **#2 All-cause mortality** | | |
| Demographics | Deceased | Deceased |
| **#3 Acute respiratory failure** | |  |
| Diagnosis | UMLS:ICD10CM:J96.0 | Acute respiratory failure |
| **#4 Hospitalization** | | |
| Visit | UMLS:HL7V3.0:VisitType:ACUTE | Visit: Inpatient Acute |
| Visit | UMLS:HL7V3.0:VisitType:IMP | Visit: Inpatient Encounter |
| Visit | UMLS:HL7V3.0:VisitType:NONAC | Visit: Inpatient Non-acute |
| Visit | UMLS:HL7V3.0:VisitType:SS | Visit: Short Stay |
| **#5 Emergency department (ED) visit** | |  |
| Visit | UMLS:HL7V3.0:VisitType:EMER | Visit: Emergency |
| **#6 Hospitalization or ED visit** | |  |
| (Have either #5 or #6) |  |  |
| **#7 Systemic glucocorticoid use** | |  |
| Medication | NLM:VA:HS051 | GLUCOCORTICOIDS (Route: Oral Product or Injectable Product) |
| **#8 Mechanical ventilation** | | |
| Procedure | UMLS:SNOMED:112798008 | Insertion of endotracheal tube |
| Procedure | UMLS:CPT:31500 | Intubation, endotracheal, emergency procedure |
| Procedure | UMLS:SNOMED:1149092001 | Positive pressure ventilation |
| Procedure | UMLS:ICD10PCS:5A1945Z | Respiratory Ventilation, 24-96 Consecutive Hours |
| Procedure | UMLS:ICD10PCS:5A1935Z | Respiratory Ventilation, Less than 24 Consecutive Hours |
| Procedure | UMLS:ICD10PCS:5A1955Z | Respiratory Ventilation, Greater than 96 Consecutive Hours |
| Procedure | UMLS:ICD10PCS:5A0945Z | Assistance with Respiratory Ventilation, 24-96 Consecutive Hours |
| Procedure | UMLS:ICD10PCS:5A0935Z | Assistance with Respiratory Ventilation, Less than 24 Consecutive Hours |
| Procedure | UMLS:ICD10PCS:5A0955Z | Assistance with Respiratory Ventilation, Greater than 96 Consecutive Hours |
| Procedure | UMLS:ICD10PCS:5A09457 | Assistance with Respiratory Ventilation, 24-96 Consecutive Hours, Continuous Positive Airway Pressure |
| Procedure | UMLS:ICD10PCS:5A09458 | Assistance with Respiratory Ventilation, 24-96 Consecutive Hours, Intermittent Positive Airway Pressure |
| Procedure | UMLS:ICD10PCS:5A09357 | Assistance with Respiratory Ventilation, Less than 24 Consecutive Hours, Continuous Positive Airway Pressure |
| Procedure | UMLS:ICD10PCS:5A09557 | Assistance with Respiratory Ventilation, Greater than 96 Consecutive Hours, Continuous Positive Airway Pressure |
| Procedure | UMLS:ICD10PCS:5A09358 | Assistance with Respiratory Ventilation, Less than 24 Consecutive Hours, Intermittent Positive Airway Pressure |
| Procedure | UMLS:ICD10PCS:5A09558 | Assistance with Respiratory Ventilation, Greater than 96 Consecutive Hours, Intermittent Positive Airway Pressure |
| Procedure | UMLS:ICD10PCS:0BH1 | Respiratory System / Insertion / Trachea |
| Procedure | UMLS:ICD10PCS:5A1935Z | Respiratory Ventilation, Less than 24 Consecutive Hours |
| Procedure | UMLS:ICD10PCS:5A1945Z | Respiratory Ventilation, 24-96 Consecutive Hours |
| Procedure | UMLS:ICD10PCS:5A1955Z | Respiratory Ventilation, Greater than 96 Consecutive Hours |

| **Supplementary Table 4.** Codes utilized in the definition of the subgroup analysis | | |
| --- | --- | --- |
| **Category** | **Code** | **Description** |
| **#1 Non-eosinophilic subgroup 1: patients with eosinophil counts consistently below 100 cells/μl** | | |
| (must not have #1.1 and must have #1.2 the following Jan 1, 2012 and Nov 1, 2020) | | |
| #1.1 |  |  |
| laboratory | TNX:LG32849-8 | Eosinophils [#/volume] in Blood (at least 0.10 10*3/uL) |
| #2.2 |  |  |
| laboratory | TNX:LG32849-8 | Eosinophils [#/volume] in Blood (between 0.00 and 0.10 10*3/uL) |
| **#2 Non-eosinophilic subgroup 2: patients with eosinophil counts above 100 cells/μl but never exceeding 300 cells/μl.** | | |
| (must not have #2.1 and must have #2.2 the following Jan 1, 2012 and Nov 1, 2020) | | |
| #2.1 |  |  |
| laboratory | TNX:LG32849-8 | Eosinophils [#/volume] in Blood (at least 0.30 10*3/uL) |
| #2.2 |  |  |
| laboratory | TNX:LG32849-8 | Eosinophils [#/volume] in Blood (between 0.10 and 0.30 10*3/uL) |

**Supplementary Table 5**. Instance level analysis table

| **Outcomes** | **Study group (S)** | **Control group (C)** | **Instance level analysis (t test)** | | | | | | |  |
| --- | --- | --- | --- | --- | --- | --- | --- | --- | --- | --- |
|  |  |  | **S Mean±SD** | **C Mean±SD** | **S Median** | **C Median** | **t** | **df** | ***p-*value** | |
| Acute exacerbation | EOS <300 cells/μL | EOS ≥ 300 cells/μL | 3.5 ± 4.8 | 3.8 ± 5.9 | 2 | 2 | -1.431 | 3220 | 0.152 | |
|  | EOS 100-299 cells/μL | EOS ≥ 300 cells/μL | 3.6 ± 5.0 | 3.8 ± 6.2 | 2 | 2 | -0.84 | 2691 | 0.401 | |
|  | EOS <100 cells/μL | EOS ≥ 300 cells/μL | 2.9 ± 3.7 | 3.6 ± 4.2 | 1 | 2 | -3.425 | 1347 | 0.001 | |
|  | EOS <100 cells/μL | EOS 100-299 cells/μL | 2.8 ± 3.7 | 3.6 ± 5.4 | 1 | 2 | -2.945 | 1159 | 0.003 | |
| Acute respiratory failure | EOS <300 cells/μL | EOS ≥ 300 cells/μL | 2.8 ± 3.7 | 3.2 ± 4.9 | 2 | 2 | -2.522 | 2345 | 0.012 | |
|  | EOS 100-299 cells/μL | EOS ≥ 300 cells/μL | 2.8 ± 3.9 | 3.4 ± 6.5 | 2 | 2 | -2.24 | 1968 | 0.025 | |
|  | EOS <100 cells/μL | EOS ≥ 300 cells/μL | 2.4 ± 2.9 | 3.0 ± 3.8 | 2 | 2 | -2.762 | 908 | 0.006 | |
|  | EOS <100 cells/μL | EOS 100-299 cells/μL | 2.4 ± 3.0 | 2.9 ± 4.9 | 2 | 2 | -1.665 | 713 | 0.096 | |
| Hospitalization | EOS <300 cells/μL | EOS ≥ 300 cells/μL | 5.3 ± 11.4 | 5.3 ± 11.6 | 2 | 2 | -0.046 | 5758 | 0.963 | |
|  | EOS 100-299 cells/μL | EOS ≥ 300 cells/μL | 6.0 ± 12.7 | 5.1 ± 10.6 | 2 | 2 | 2.459 | 4852 | 0.014 | |
|  | EOS <100 cells/μL | EOS ≥ 300 cells/μL | 3.6 ± 6.7 | 5.1 ± 9.1 | 2 | 2 | -4.344 | 2388 | < 0.001 | |
|  | EOS <100 cells/μL | EOS 100-299 cells/μL | 3.7 ± 6.8 | 5.7 ± 12.2 | 2 | 2 | -4.811 | 2146 | < 0.001 | |
| ED visit | EOS <300 cells/μL | EOS ≥ 300 cells/μL | 2.5 ± 2.9 | 2.8 ± 3.5 | 2 | 2 | -3.23 | 5166 | 0.001 | |
|  | EOS 100-299 cells/μL | EOS ≥ 300 cells/μL | 2.6 ± 3.2 | 2.8 ± 3.4 | 2 | 2 | -1.443 | 4389 | 0.149 | |
|  | EOS <100 cells/μL | EOS ≥ 300 cells/μL | 2.2 ± 1.9 | 2.8 ± 3.5 | 1 | 2 | -4.847 | 2084 | < 0.001 | |
|  | EOS <100 cells/μL | EOS 100-299 cells/μL | 2.2 ± 2.0 | 2.7 ± 3.7 | 1 | 2 | -3.437 | 1908 | 0.001 | |
| Hospitalization or ED visit | EOS <300 cells/μL | EOS ≥ 300 cells/μL | 5.3 ± 10.5 | 5.6 ± 10.8 | 2 | 3 | -1.288 | 7590 | 0.198 | |
|  | EOS 100-299 cells/μL | EOS ≥ 300 cells/μL | 5.8 ± 11.6 | 5.5 ± 10.0 | 2 | 3 | 1.431 | 6441 | 0.152 | |
|  | EOS <100 cells/μL | EOS ≥ 300 cells/μL | 3.9 ± 6.2 | 5.4 ± 9.0 | 2 | 3 | -5.668 | 3143 | < 0.001 | |
|  | EOS <100 cells/μL | EOS 100-299 cells/μL | 3.9 ± 6.3 | 5.7 ± 11.4 | 2 | 3 | -5.163 | 2898 | < 0.001 | |
| Mechanical ventilation | EOS <300 cells/μL | EOS ≥ 300 cells/μL | 1.6 ± 1.3 | 1.7 ± 1.6 | 1 | 1 | -1.264 | 1559 | 0.206 | |
|  | EOS 100-299 cells/μL | EOS ≥ 300 cells/μL | 1.7 ± 1.4 | 1.8 ± 1.8 | 1 | 1 | -1.067 | 1304 | 0.286 | |
|  | EOS <100 cells/μL | EOS ≥ 300 cells/μL | 1.5 ± 1.2 | 1.7 ± 1.6 | 1 | 1 | -2.102 | 591 | 0.036 | |
|  | EOS <100 cells/μL | EOS 100-299 cells/μL | 1.5 ± 1.3 | 1.5 ± 1.2 | 1 | 1 | -0.322 | 475 | 0.748 | |

EOS: Eosinophil count; ED: emergency department; SD: standard deviation; t: t-statistic; df: degrees of freedom.

**Supplementary Table 6**. Hazard ratio and incidence for comparing matched subgroups for each outcome

| **Outcomes** | **Subgroup** **Eosinophil <100** Event No. (%) | **Subgroup**  **Eosinophil 100-299** Event No. (%) | **HR (95%CI)** | ***P*-value** |
| --- | --- | --- | --- | --- |
| **Primary outcome** |  |  |  |  |
| Acute exacerbation | 548 (18.8) | 613 (21.1) | 0.932 (0.831,1.046) | 0.232 |
| **Secondary outcomes** |  |  |  |  |
| All-cause mortality | 462 (15.9) | 511 (17.6) | 0.950 (0.837,1.077) | 0.422 |
| Acute respiratory failure | 337 (11.6) | 378 (13.0) | 0.939 (0.811,1.088) | 0.404 |
| **Other outcomes** |  |  |  |  |
| Hospitalization | 1,024 (35.2) | 1,124 (38.6) | 0.940 (0.863,1.023) | 0.149 |
| ED visit | 897 (30.8) | 1,013 (34.8) | 0.909 (0.831,0.994) | 0.037 |
| Hospitalization or ED visit | 1,388 (47.7) | 1,512 (51.9) | 0.926 (0.861,0.996) | 0.040 |
| Systemic glucocorticoid use | 1,096 (37.7) | 1,049 (36.0) | 1.108 (1.018,1.206) | 0.017 |
| Mechanical ventilation | 215 (7.4) | 262 (9.0) | 0.861 (0.719,1.032) | 0.104 |

CI: confidence interval; HR: hazard ratio; ED: emergency department.

**Supplementary Table 7.** Hazard ratio and incidence for comparing matched inhalant subgroups for primary outcome

| Primary outcome | **Acute exacerbation** | | | | | | | |
| --- | --- | --- | --- | --- | --- | --- | --- | --- |
|  | Non-eosinophilic group | | Eosinophilic group | | HR (95%CI) | | *P* value | |
|  | Event No. (%) | | Event No. (%) | |  |  |  |  |
| LABA or LAMA | | 117 (13.8) | | 128 (15.1) | | 0.89 (0.69,1.14) | | 0.3586 |
| LABA + LAMA | | 42 (18.9) | | 38 (17.1) | | 1.12 (0.72,1.74) | | 0.6052 |
| LABA+ ICS | | 165 (22.3) | | 186 (25.2) | | 0.85 (0.69,1.05) | | 0.1391 |
| LABA + LAMA + ICS | | 1,237 (43.5) | | 1,380 (48.5) | | 0.86 (0.80,0.93) | | 0.0001 |

CI: confidence interval; HR: hazard ratio; ED: emergency department; LABA: long‐acting beta‐agonists; LAMA: long‐acting muscarinic antagonists; ICS: inhaled corticosteroid.

**Supplementary Table 8.** Hazard ratio and incidence for comparing matched inhalant subgroups for secondary outcomes

| Secondary outcomes | **All-cause mortality** | | | | | | **Acute respiratory failure** | | | |
| --- | --- | --- | --- | --- | --- | --- | --- | --- | --- | --- |
|  | Non-eosinophilic group | Eosinophilic group | | HR (95%CI) | | *P* value | Non-eosinophilic group | Eosinophilic group | HR (95%CI) | *P* value |
|  | Event No. (%) | Event No. (%) | |  |  |  | Event No. (%) | Event No. (%) |  |  |
| LABA or LAMA | 211 (24.9) | 243 (28.7) | 0.85 (0.70,1.02) | | 0.0722 | | 117 (13.8) | 163 (19.2) | 0.69 (0.54,0.88) | 0.0021 |
| LABA + LAMA | 38 (17.1) | 39 (17.6) | 0.98 (0.63,1.54) | | 0.9434 | | 23 (10.4) | 23 (10.4) | 1.00 (0.56,1.79) | 0.9894 |
| LABA+ ICS | 191 (25.8) | 241 (32.6) | 0.77 (0.64,0.94) | | 0.0077 | | 149 (20.2) | 207 (28.0) | 0.69 (0.56,0.86) | 0.0006 |
| LABA + LAMA + ICS | 590 (20.7) | 670 (23.5) | 0.88 (0.79,0.99) | | 0.0253 | | 782 (27.5) | 871 (30.6) | 0.88 (0.80,0.97) | 0.0088 |

CI: confidence interval; HR: hazard ratio; LABA: long‐acting beta‐agonists; LAMA: long‐acting muscarinic antagonists; ICS: inhaled corticosteroid.
